# *In-office* bacteria test for a microbial monitoring during the conventional and self-ligating orthodontic treatment

**DOI:** 10.1186/1746-160X-9-7

**Published:** 2013-02-01

**Authors:** Stefano Mummolo, Enrico Marchetti, Maria Rita Giuca, Gianni Gallusi, Simona Tecco, Roberto Gatto, Giuseppe Marzo

**Affiliations:** 1 Department of MeSVA, University of L’Aquila, L’Aquila, Italy; 2 Department of “Patologia Chirurgica, Medica, Molecolare e dell'Area Critica”, University of Pisa, Pisa, Italy; 3 School of Orthodontics, University of L’Aquila, L’Aquila, Italy; 4 Home address:, Via Le Mainarde 26, Pescara 65124, Italy

## Abstract

This study investigated the microbial level of Streptococcus mutans and Lactobacillus spp. during an orthodontic treatment, and compare the data with untreated control subjects.

Sixty young adult subjects were selected (average 20.5, DS 1.62), among which 40 underwent an orthodontic treatment (20 were treated with self-ligating brackets and 20 with conventional brackets) and 20 were controls. Plaque Index, salivary flow and buffering capacity of saliva were assessed before the beginning of the orthodontic treatment. Then the microbial counts were obtained by using an *in-office* bacteria test.

The plaque index (PI) increased over time in each group as well as salivary flow, mostly in subjects treated with self-ligating brackets, suggesting a difference between conventional and self-ligating brackets. *S.mutans* showed a different trend of colonization in the two treated groups, as for subjects treated with conventional brackets it showed the greater value at the early stage of treatment (T1), followed by a decrease at T2. Lactobacillus spp. showed significant increase over time in the two treated groups, respect to the control group. Linear regression analysis showed no significant predictor for the microbial count at T2.

The assortment of the various species of bacteria change over time during the orthodontic treatment, and seems to show different trends, depending on the type of orthodontic device. Consequently a periodical microbial monitoring using *in-office* bacteria tests, seems indicated.

## Introduction

For the prevention of caries during the orthodontic treatment, a clear dominance of *S.mutans* alone is not decisive for a high caries risk. *Lactobacillus spp.* alone or the combination of *S.mutans* and *Lactobacillus spp.* come into play. Consequently, the occurrence of both types of bacteria must be evaluated [1]. For the S.mutans, the pre-treatment levels of S. mutans seem to be significant predictors of the levels of S. mutans after placement of orthodontic appliances, while this was not the case for total bacterial counts.

In general, the determination of both the *S.mutans* and the *Lactobacillus spp.* seems to increase the accuracy of the prognosis, as the two species have different distribution and characteristics linked to the carie [2,3]:

S. mutans levels are elevated in many healthy subjects, and so there is a low specificity for S. mutans as a predictive test for caries. The percent S. mutans is high in the biofilms over white spots and even higher over cavitary lesions, consistent with a recognized, key role in caries [4].

*Lactobacillus spp.* are mainly responsible for caries progression, i.e. they cause active damage to the tooth structure by multiplying and spreading the bacteria. They are characterized by the following properties: colonization in retention niches, acid production, acid tolerance and are indicators of high sugar intake. They are more resistant to bacteria-reducing substances, such as chlorhexidine, than mutans streptococci and are usually found in areas that are difficult to clean, as for example near to orthodontic devices. Therefore, it is not surprising that there is a significant correlation between carious lesions and the lactobacillus spp. count in both adults and children [5].

The aim of this study was to investigate in-office bacteria test for a microbial monitoring during the conventional and self-ligating orthodontic treatment. In this study the levels of S.mutans and Lactobacillus spp. were evaluated in the oral saliva of patients orthodontically treated with self-ligating brackets or conventional brackets and then compared with those of an untreated control group, before the beginning of treatment and after three and six months.

## Material and methods

The individuals participating in the study group were selected from a larger pool of patients from the practice of one author (SM), using the following inclusion criteria: young adult subjects treated with fixed appliances, no reported oral habits detrimental to health, including smoking, absence of restorations, and/or missing teeth due to dental caries. There were no stories of periodontal diseases as gingival inflammation among the subjects.

The patients who fulfilled these criteria were randomly assigned to one of the treatment groups. Groups were homogeneous in the gender distribution and age distribution. Demographic data (age) and data about PI, buffering capacity of saliva and salivary flow are reported in Table 1.

**Table 1:** Demographic (age, range for each group) and oral and saliva characteristics (PI, salivary flow, buffering capacity of saliva) of the samples at T0

**Variable**	**Self-ligating brackets group**	**Conventional brackets group**	**Control group**
Age (years)	(range: 18–23 years) average 20.5, DS 1.62	(range: 18–23 years) average 19.8, DS 1.3	(range: 18–23 years) average 20.2, DS 2.1
PI (T0) ^1^	0	0	0
Salivary flow ^2^	59	51	50
	(Sum of the individual scores at T0)	(Sum of the individual scores at T0)	(Sum of the individual scores at T0)
Buffering capacity of saliva ^3^	Middle capacity	Middle-high capacity	Middle capacity

^1^ For the PI variable, the scores are 0, 1, 2 and 3.

^2^ For the Salivary flow, the scores are: 1=<1; 2=1<x<1.5; 3=1.5<x<1.75; 4=1.75<x<2; 5=2<x<2.5; 6>2.5.

^3^ For the Buffering capacity of saliva, the scores are: 1=low; 2=middle-low; 3=middle; 4=middle-high; 5=high.

The orthodontist did not know the bracket group assignment at the time of the first saliva collection.

Consent for saliva collection was obtained from all patients.

The 60 subjects (27 male and 33 female) were divided into three equal groups. 20 patients were treated with in-ovation GAC self-ligating brackets(ditta) ; 20 patients were treated with ovation GAC conventionally ligated brackets according to the Roth technique and 20 patients were considered the control untreated group.

For the numerosity of the samples, we applied the formula [6]

(1) $$N=t^{2}\;P\;\left(1-P\right)/\alpha^{2}$$

Where N is the numerosity of the sample, t is the t distribution; P is the expected prevalence (in this case 10%).

We considered a normal value of S.mutans as <10^5^ and a mean difference of approximately 10% level as clinically significant difference between the study and the control group, in the prevalence of subjects with *S. mutans* counts > 10^5^. The same difference was also applied to the *Lactobacillus spp.* counts.

Therefore considering α= 0.06, the sample size requested was 20 subjects.

Before beginning the study, standard oral hygiene instructions were provided (the modified Bass technique), with specific attention to the orthodontic appliances, and also to control subjects.

We recommended the modified Bass technique and the use of dental Superfloss, three times in a day, and a mouthwash with fluoride and chlorhexidine 0.05 before going to bed. We also ordered a session of professional hygiene before the beginning of the study.During the study, all patients were highly motivated to diligent home oral hygiene.

Whole stimulated saliva was collected from each patient at three time points before bonding and initiation of orthodontic therapy (baseline at T0), at a period of 3 months (T1) after full bonding had taken place, and after 6 months from T0 (T2).

The subjects had refrained from eating or drinking beverages, or brushing teeth for at least 1 hour before saliva collection, as indicated by the manufacturer of the in-office kit, as all these actions would have affected the mean flow of saliva.

Collection of saliva samples was performed before any oral examination or manipulation of the orthodontic device, so as not to disrupt the oral microbiota.

At each time point, each patient was given a tablet of paraffin-stimulating saliva that had to be chewed for 30 seconds, and then was asked to remove the produced saliva.

Then, the tablet of paraffin-stimulating saliva was re-administered, asking to the patient to gum it for 30 seconds, while collecting the produced saliva in a graduated small glass during the following 5 minutes.

### Plaque index (PI)

The PI as developed by Silness and Loe assesses the thickness of plaque at the cervical margin of the tooth (closest to the gum). Four areas, distal, facial or buccal, mesial, and lingual, are examined.

Each tooth is dried and examined visually using a mirror, an explorer, and adequate light. The explorer is passed over the cervical third to test for the presence of plaque. A disclosing agent may be used to assist evaluation. Four different scores are possible. A zero indicates no plaque present; 1 indicates a film of plaque present on the tooth; 2 represents moderate accumulation of soft deposits in the gingival pocket or on the tooth that can be seen by the naked eye; 3 represents an abundance of soft matter within the pocket or on the tooth. Each area of each tooth is assigned a score from 0 to3. Scores for each tooth are totaled and divided by the four surfaces scored. To determine a total PI for an individual, the scores for each tooth are totaled and divided by the number of teeth examined. Four ratings may then be assigned: 0 = excellent, 0.1-0.9 = good, 1.0-1.9 = fair, 2.0-3.0 = poor

For each participant, the plaque index (PI) was assessed, considering the percentage of surfaces with plaque (taking into consideration all surfaces per tooth for all erupted teeth). The index was recorded after each saliva sample collection at each visit without the use of a plaque disclosing agent.

The procedure was standardized and its repeatability was evaluated with the Intraclass Correlation Coefficient (ICC) applied to double measurement recorded from 5 subjects two times, at a distance of 30 minutes between the first and the second evaluations.

### Assessment of S. mutans and Lactobacillus spp. colonization

The saliva samples were inoculated on two different selective agar plates, one for *S. mutans* and the other for *Lactobacillus spp.* to record the count of colonies.

The CRT bacteria test from Ivoclar Vivadent enables the simultaneous determination of the *S. mutans* and *Lactobacillus spp.* counts in saliva by means of selective agars. The blue mitis-salivarius-agar with bacitracin was used to detect mutans streptococci, while the light culture medium, Rogosa agar, was used to evaluate *Lactobacillus*. Foils protected the agars from drying out and contamination. The deep indentation in the carriers prevented the culture media from slipping out.

The agar plates were incubated at 37°C for 2 days in a CO2 atmosphere (CRT incubator), after added with a tablet of NaHCO_3_ to stimulate bacterial growth, following which the total count was performed. The NaHCO_3_ tablet placed in the test vial releases CO2 when it comes into contact with moisture. This creates favorable conditions for bacterial growth.

*S.mutans* occurred as small blue colonies with a diameter of < 1 mm on the blue agar, while *Lactobacillus spp.* were detected as white colonies on the transparent agar. The comparison with the corresponding pictures in the model chart permitted the assessment of the caries risk. In this context, counts higher than 10^5^ CFU of *S. mutans* and/ or *Lactobacillus* per milliliter of saliva indicating a high/lower risk for dental caries [7], were recorded.

All laboratory procedures were carried out without the personnel knowing the allocation of saliva samples to bracket groups.

### Assessment of salivary flow

After administration of the tablet of paraffin, the subjects were asked to chew it for 30 seconds, while all the saliva produced during the following 5 minutes was collected in a graduated glass. So the salivary flow was recorded, calculated as the milliliters of saliva produced during a certain period of time (five minutes).

### Assessment of the buffering capacity of saliva

The CRT^®^ buffer (Caries Risk Test, Ivoclar, Vivadent) was used to determine the buffer capacity of saliva by means of a test strip featuring a special indicator system. It was determined as low, middle or high power.

### Study on method error

In order to verify the calibration of the procedure, a preliminary study on method error was performed; to 5 subjects measurements were taken at two times by the same operator before the beginning of the study. The data were collected and compared with the Chi-square test (for the nominal variables) and with the Intra-Class correlation coefficient to assess the repeatability of the procedure.

### Statistical analysis

Demographic and clinical characteristics of the sample were investigated with conventional descriptive statistics. Differences at T0 were evaluated with Chi-square test for the distribution between males and females and for the prevalence of subjects with bacterial count > 10^5^. Non parametric tests (Friedman test) were used to assess inter-group differences for the other variables, followed by the Mann–Whitney U test as post-hoc analyses (for the variables PI, buffering capacity of saliva, and salivary flow). It was used also the Friedman test which is a non-parametric test (distribution-free) used to compare observations repeated on the same subjects. This is also called a non-parametric randomized black analysis of variance. The Wilcoxon signed rank test was used to assess intra-gruoup differences.

The percentages of subjects with a total count of *S. mutans* <105 at T1 and T2 were then analyzed with linear regression analysis with the count of *S. mutans* (or *Lactobacillus spp.*) as dependent variables, and using initial bacterial counts or salivary flow, or buffering capacity of saliva, as the independent variables.

Simple linear regression analysis was used to evaluate the initial levels of total bacteria counts and *S. mutans* as predictors of the levels of these at the second time interval. Simple linear regression analysis was also studied to evaluate the effect of the saliva buffering capacity, the salivary flow, and the initial or subsequent PI value.

Data analysis was conducted with the statistical package, PSPP (psppire data editor 07.7 – gfb5c91; copyright 2007 Free Software Foundation, Inc.) for MAC Pro X, at the 0.05 level of significance.

## Results

No difference was found in the demographics and oral hygiene indices between the two groups, verifying the random assignment of brackets to the population sample.

Table 1 shows the distribution of demographic, oral and saliva variables, for the three groups at T0. The distribution of gender and age did not show any difference among the three groups., as also the variables concerning the PI, the salivary flow and the buffering capacity of saliva, also showed in the table.

The PI data in the three groups over the time are reported in Table 2. The PI value increased over time in all the three groups, with a statistically significant relevance between T2 and T0.

**Table 2:** Main results

**Plaque Index (PI)**	**PI (T0)**	**PI (T1)**	**PI (T2)**	**T0 versus T1**	**T0 versus T2**	**T1 versus T2**
Self-ligating brackets group	0	23	28	W= 153.06 P=0.001	W= 97.53 P=0.001	NS
Conventional brackets group	0	33	37 **	W= 133.06 P=0.001	W= 178.0 P=0.001	NS
Control group	0	14 **	27	W= 127.88 P=0.001	W= 188.0 P=0.001	NS
	**Buffering capacity (indicated as the more frequent nominal value for each group)**			**Self-ligating group VERSUS Control group**	**Conventional group VERSUS Control group**	**Self-ligating group VERSUS Cnventional group**
Self-ligating brackets group	3 (middle boffering capacity)					NS
Conventional brackets group	4 (middle-high capacity)			NS		
Control group	3 (middle capacity)				NS	
	**Salivary Flow (T0)**	**Salivary Flow (T1)**	**Salivary Flow (T2)**	**T0 versus T1**	**T0 versus T2**	**T1 versus T2**
Self-ligating brackets group	59	69	69	W= 10.7 P=0.05	W= 97.53 P=0.001	NS
Conventional brackets group	51	59	59	NS	NS	NS
Control group	50	50 **	50 *	NS	NS	NS
	**% of subjects with abnormal value (>10**^**5**^**) T0**	**% of subjects with abnormal value (>10**^**5**^**) T1**	**% of subjects with abnormal value (>10**^**5**^**) T2**	**T0 versus T1**	**T0 versus T2**	**T1 versus T2**
Self-ligating brackets group	0%	25%	45%	NS	Chi-square= 10.17 P= 0.01	NS
Conventional brackets group	0%	60% **	20% **	Chi-square = 97.53 P=0.001	Chi-square= 24.47 P= 0.001	Chi-square= 88.6 P=0.001
Control group	0%	5%	35%	NS	Chi-square= 10.75 p=0.001	Chi-square= 14.25 P=0.01
	**% of subjects with abnormal value (>10**^**5**^**) T0**	**% of subjects with abnormal value (>10**^**5**^**) T1**	**% of subjects with abnormal value (>10**^**5**^**) T2**	**T0 versus T1**	**T0 versus T2**	**T1 versus T2**
Self-ligating brackets group	0%	10%	25%	Chi-square=5 P=0.03	Chi-square=4.27 P=0.04	Chi-square=27.22 P=0.001
Conventional brackets group	0%	50%	70%	Chi-square=35.5 P=0.001	Chi-square=80 P=0.001	NS
Control group	0%	10%	30%	NS	Chi-square=8.8 P=0.001	Chi-square=8.8 P=0.001

The total PI (Plaque Index) for each group, expressed as the sum of the individual scores for each group. Significant differences among the three groups were assessed with the Friedman test for each group and the Mann-Whithney U test to assess inter-group differences and Wilcoxon signed rank test (W) for intra-group differences. Buffering capacity of saliva, expressed as the more frequent evaluation in each group, recorded before the beginning of the study. Significant differences were assessed with Chi-square test comparing five categories (low, middle-low, middle, middle-high, and high). Salivary flow expressed as the sum of the single rates for each subject. Significant differences were assessed with Friedman test and the Mann–Whitney U test and the Wilcoxon signed rank test (W) for inter-groups and intra-group differences, respectively. Prevalence of subjects with abnormal values of microbial colonization calculated as percentage respect to the numerosity of each group.

At T2, subjects treated with conventional brackets showed a significant higher value of PI respect to the other two groups.

The Table 2 shows the data of the buffering capacity of saliva in the three groups. No significant difference was observed in the buffering capacity of saliva among the three groups.

Table 2 shows the results for the salivary flow at T0, T1 and T2 for the three groups. Comparisons were made through the Chi-square test. Subjects treated with self-ligating brackets showed a significant higher salivary flow respect to the other groups at T2, while no significant difference was observed at T0.

The prevalence of subjects with an abnormal concentration of *S.Mutans* (>10^5^) or *Lactobacillus spp.* (>10^5^) was expressed as percentage in the whole group of subjects. The data are reported in Table 2 for the *S. Mutans* and for the *Lactobacillus spp*. *S.mutans* seems to show a different trend of colonization as it showed the great value at the early stage of the orthodontic treatment (3 months after the beginning, T1) and then a significant decrease, registered at T2, after six months.

For the Lactobacillus spp. (Table 2), statistical analysis showed differences between the two treated groups at either T1 or T2, as patients treated with conventional brackets showed statistically significant higher prevalence of colonization, respect to subjects treated with self-ligating brackets.

In the analysis of simple linear regression (Table 3), the three variables used as independent variables had a significant effect on the count of *Lactobacillus spp.*, but when analyzed separately, the effect was not significant. No significant linear relationship was observed for the *S. mutans.*

**Table 3:** Linear regression analysis using PI, Buffering capacity of saliva and salivary flow as independent variables

	**B**	**St.Error**	**Beta**	**t**	**Significance**
(Constant)	2.11	0.33	0.00	6.46	0.001 **
PI	−0.23	0.16	−0.32	6.46	0.16
Buffering capacity	−0.02	0.8	−0.07	−0.32	0.75
Salivary flow	−0.1	0.06	−0.4	−1.74	0.1

## Discussion

The objective of this study was to investigate the microbial level of Streptococcus mutans and Lactobacillus spp. during the initial phases of an orthodontic treatment, and compare the data with untreated control subjects. To our knowledge, the comparison of CRT bacteria test, used in this study, with laboratory methods showed a convincing correlation in literature [8]. In this study, the adhesion of bacteria to the brackets surface was not evaluated, but simply the percentage of subjects with abnormal microbial colonization in their saliva, as salivary level of microbial reflects the variation of those attached to bonded teeth. The assessment of salivary microbial counts may not be related to the actual adhered microbia onto hard dental surfaces. Of course the use of a parafinwax to stimulate salivary flow may reduce the weight of the effect by detaching microbia from surfaces.

The PI, the salivary flow and the buffering capacity of saliva were also evaluated, in order to assess their role as significant predictors of the microbial colonization over time. On the base of our results, the assortment of the various species of bacteria in the oral microbial population observed during the early stages of the orthodontic treatment is not necessarily the same as that observed after a few months of orthodontic treatment. In addition, the microbial assortment seems to show different trends, depending on the type of orthodontic device. In this study, conventional and self-ligating brackets were compared. Self-ligating brackets demonstrated low friction during the early phases of orthodontic alignment, respect to conventional brackets [9]. PI, salivary flow, buffering capacity of saliva, or data on microbial colonization conducted at the early stage of orthodontic treatment are not adequate predictors of the assortment of microbial species in the oral saliva during the following stages of orthodontic treatment. Consequently, a continuous microbial monitoring, performed by dentists using *in-office* bacteria tests, seems to be indicated during the orthodontic treatment, in order to improve the preventive approach to intra-oral diseases.

The data observed for the variables evaluated (PI, buffering capacity of saliva and salivary flow) suggested various clinical factors.

For example, despite the fact that subjects wearied an orthodontic fixed devices, no statistically significant differences in the trend of PI were observed between the test and the control groups, as PI increased in all the three groups during the follow-up. This observation suggests that the efficacy of home hygiene instructions is long only for a few months, after which, new motivation of subjects is needed on their oral hygiene, despite the wearing of orthodontic appliances.

About the results regarding the buffering capacity of saliva, no significant differences among the three groups were observed, as expected, while the salivary flow showed a significant increase over time in the treated and control groups, mostly in the group of patients treated with self-ligating brackets respect to the control subjects. This could be related to the different design of self-ligating brackets [10].

For the microbial count, it is note that caries-free subjects usually present with < 10^5^ CFU of *S. mutans* per milliliter of saliva. ^7^ This level was considered the cut-off value to record the data.

**Figure 1:**
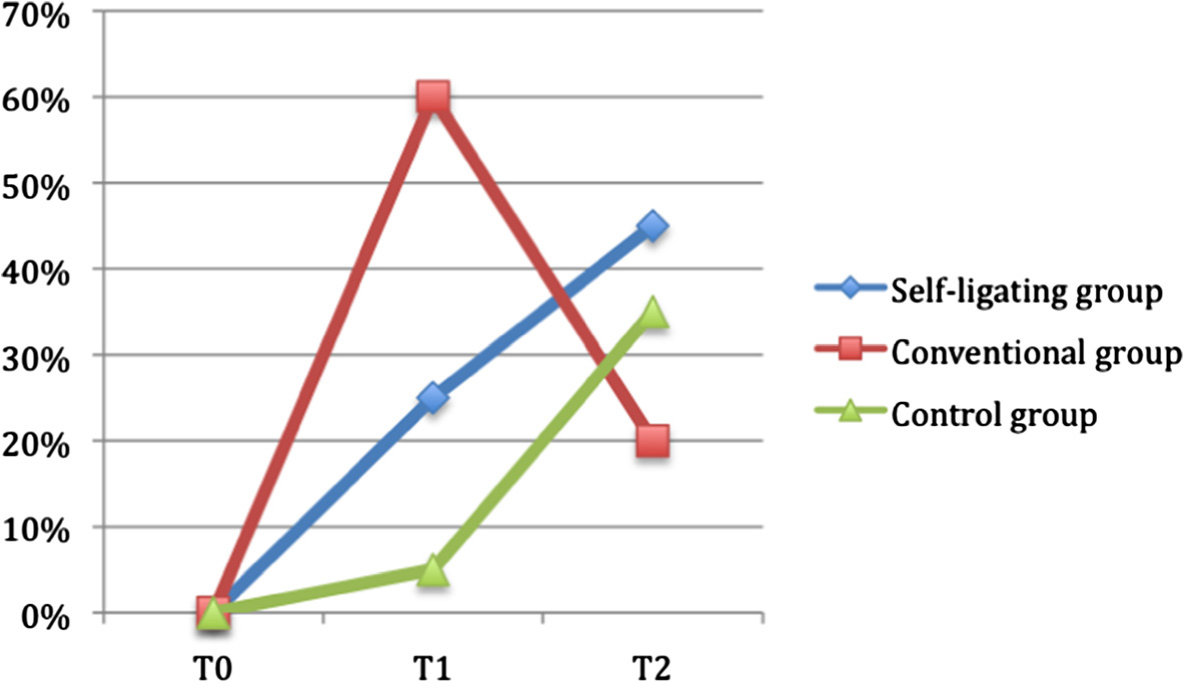
**Percentage of subjects with S.mutans colonization >10**^**5**^**evaluated at T0, T1 and T2.** See text for details.

In this study a different trend in the microbial colonization for the two treated groups was observed, as the group treated with conventional appliances showed a drastic increase of subjects with *S. mutans* counts >10^5^ after 3 months from the beginning of treatment (60%), with a significant reduction after other 6 months, when they showed lower values respect to the other two groups (20%) (Table 2 and Figure 1). As the PI index showed about the same trend in the three groups, the different trend in the *S. mutans* colonization seems not to be associated to the accumulation of plaque, but rather to different affinity for elastomeric ligations (used with conventional brackets) of the two examined species. The elastomeric ligatures coupled with conventional brackets are monthly changed and therefore may have an effect on bacterial attachment since the substrate for colonization is eliminated and a new cycle of colony formation is initiated.

Türkkahraman *et al*. (2005) [11] examined the effect of two ligation modes (elastomeric rings and ligatures wires) on microbial accumulation before therapy and found slightly higher total counts of bacteria after 5 weeks of treatment around the elastomeric rings. Bacteria can be less or more attracted by different surfaces. For example, it was stated that surface-free energy such as *S. mutans* should prefer surfaces with high surface-free energy materials such as stainless steel brackets [12].

In subjects wearing a conventional appliance, the level of *S. mutans* at the early stages of orthodontic treatment (3 months) resulted not to be predictive of the future presence of *S. mutans* colonization, and seemed not to follow the increasing trend of the plaque accumulation in the same subjects; these results seem to respond to natural biological changes in the general assortment of oral microbial population, which could be probably associated to the presence of elastic ligatures in the mouth. However, the sudden significant reduction in the prevalence of *S. mutans* colonization in the conventional group at T2 resulted not in line with the general trend of the other variables studied in this research, which showed a considerable overlap in all the three groups (see the data about PI and salivary flow). *Lactobacillus spp.* showed a similar trend characterized by a constant increase in the two study groups, and also, at a lower level, in the control group.

For the subjects treated with conventional brackets, the different trends of these two bacteria seem to underline different affinity of these two species for the materials of the appliances, mostly the presence of elastic ligatures.

Aim of the regression analysis performed in this study was to investigate wether saliva flow and buffering capacity of saliva can be validate predictors to assess microbial counts in the saliva after 3 or 6 months of treatment.

If this is the case, then, these variable could be used to predict the correct hygiene program and caries prevention program during an othodontic treatment.

For the *Lactobacillus spp.*, the salivary flow, PI and the buffering capacity of saliva seem to be predictive to a low rate of the microbial counts at T2, but none of these variables, considered alone, can be considered useful as a general predictor of the microbial colonization. About the buffering capacity, the low capacity seems to promote bacterial colonization, and the same seems to be verified for the salivary flow. Lactobacillus spp. preferably settle and multiply in an acid environment, even at a very low pH-value of 5.2.

About saliva flow rates, it was demonstrated that at low flow rates or under static conditions, the grooves of rough surfaces of a bracket may act as stagnation points, thereby promoting biofilm maturation [13].

For the PI, we can observe an inverse relationship between the PI and the *Lactobacillus spp.* colonization, as suggested in the linear regression analysis. This observation can be explained because usually, there are only few *Lactobacillus spp.* in saliva. Their number increases if *S. mutans* start to colonize in the oral cavity, since they produce a favorable acid environment for *Lactobacillus spp.*, so the pH-value decreases [14]. *Lactobacillus spp.* preferably settle in niches with a low pH-value and in the vicinity of plaque accumulation [15]. Consequently, *Lactobacillus spp.* can be more easily found in cavities and carious dentin. In contrast to *S. mutans*, *Lactobacillus spp.* do not adhere to tooth surfaces on their own account, but need natural or iatrogenic retention niches, such as brackets, where it is usually difficult to reach and clean.

For the *S. mutans,* this study failed to demonstrate such a correlation, because the microbial counts at T1, the buffering capacity of saliva, the PI and the salivary flow were not predictive for the same bacterial count at T2.

In an examination carried out on orthodontic young adult patients, plaque index, *S. mutans* and *Lactobacillus spp.* counts, and salivary buffer capacity were significantly higher in the group treated in the public structures compared with the patients treated in the private structures [16].

Orthodontic treatment changes the oral environmental factors, promotes an increase in stimulated flow rate, buffer capacity and salivary pH, which augment the anti caries activity of saliva, [17] but that study was carried out for only one month.

Given the multi-causal nature of caries, however, no reliable, general forecast can be made by examining bacterial counts. An early control of the bacterial counts may contribute to a decrease in caries development in the long run.

## Conclusions

The aim of this study was to evaluate the microbial colonization in the mouth during an orthodontic treatment with conventional or self-ligating brackets and to assess the differences with control untreated subjects.

The assortment of the various species of bacteria in the oral microbial population observed during the early stages of the orthodontic treatment is not necessarily the same as that observed after a few months of orthodontic treatment. In addition, the microbial assortment seems to show different trends, depending on the type of orthodontic device. PI, salivary flow, buffering capacity of saliva, or data on microbial colonization conducted at the early stage of orthodontic treatment are not adequate predictors of the assortment of microbial species in the oral saliva during the following stages of orthodontic treatment. In conclusion, a continuous microbial monitoring, performed by dentists using *in-office* bacteria tests, seems to be indicated during the orthodontic treatment, in order to improve the preventive approach to intra-oral diseases.

## Competing interests

The author(s) declare that they have no competing interests.

## Authors’ contributions

SM organized the protocol of research; EM coordinated the recording of data; GG helped in the recording of data; MRG helped in the revision of literature; ST organized the data, made the statistical analysis, the analysis of results, the discussion of results, the tables, the figures and draft the manuscript; RG and GM revised the manuscript. All the authors read and approved the final manuscript.

## Authors’ information

Stefano Mummolo and Simona Tecco are principal investigator to this work.
